# Genome-wide identification and expression analysis of the *bHLH* transcription factor family and its response to abiotic stress in foxtail millet (*Setaria italica* L.)

**DOI:** 10.1186/s12864-021-08095-y

**Published:** 2021-10-30

**Authors:** Yu Fan, Dili Lai, Hao Yang, Guoxing Xue, Ailing He, Long Chen, Liang Feng, Jingjun Ruan, Dabing Xiang, Jun Yan, Jianping Cheng

**Affiliations:** 1 College of Agriculture, Guizhou University, Huaxi District, Guiyang, Guizhou Province 550025 People’s Republic of China; 2 School of Food and Biological engineering, Chengdu University, Chengdu, 610106 People’s Republic of China; 3 Department of Nursing, Sichuan Tianyi College, Mianzhu, 618200 People’s Republic of China; 4 Chengdu Institute of Food Inspection, Chengdu, 610030 People’s Republic of China

**Keywords:** Foxtail millet, *bHLH* gene family, Genome-wide analysis, Abiotic stress

## Abstract

**Background:**

Members of the basic helix-loop-helix (*bHLH*) transcription factor family perform indispensable functions in various biological processes, such as plant growth, seed maturation, and abiotic stress responses. However, the *bHLH* family in foxtail millet (*Setaria italica*), an important food and feed crop, has not been thoroughly studied.

**Results:**

In this study, 187 *bHLH* genes of foxtail millet (*SibHLHs*) were identified and renamed according to the chromosomal distribution of the *SibHLH* genes. Based on the number of conserved domains and gene structure, the *SibHLH* genes were divided into 21 subfamilies and two orphan genes via phylogenetic tree analysis. According to the phylogenetic tree, the subfamilies 15 and 18 may have experienced stronger expansion in the process of evolution. Then, the motif compositions, gene structures, chromosomal spread, and gene duplication events were discussed in detail. A total of sixteen tandem repeat events and thirty-eight pairs of segment duplications were identified in *bHLH* family of foxtail millet. To further investigate the evolutionary relationship in the *SibHLH* family, we constructed the comparative syntenic maps of foxtail millet associated with representative monocotyledons and dicotyledons species. Finally, the gene expression response characteristics of 15 typical *SibHLH* genes in different tissues and fruit development stages, and eight different abiotic stresses were analysed. The results showed that there were significant differences in the transcription levels of some *SibHLH* members in different tissues and fruit development stages, and different abiotic stresses, implying that *SibHLH* members might have different physiological functions.

**Conclusions:**

In this study, we identified 187 *SibHLH* genes in foxtail millet and further analysed the evolution and expression patterns of the encoded proteins. The findings provide a comprehensive understanding of the *bHLH* family in foxtail millet, which will inform further studies on the functional characteristics of *SibHLH* genes.

**Supplementary Information:**

The online version contains supplementary material available at 10.1186/s12864-021-08095-y.

## Background

Basic-helix-loop-helix (bHLH) transcription factors are widely present in eukaryotes. Although they are not unique to plants, they still form one of the largest transcription factor families in plants due to their numerous members [1, 2]. There is a *bHLH* domain in the *bHLH* transcription factor family, the domain sequence is highly conserved, with a total of approximately 50–60 amino acid residues. The domain comprises two functional regions: the basic region and the helix-loop-helix (HLH) region [3, 4]. The basic region is located at the N-terminus and contains approximately 15 amino acids. It binds to the cis-acting element E-box (5′-CANNTG-3′) and determines whether *bHLH* transcription factors bind to DNA [3, 4]. The HLH region is distributed at the C-terminus of the gene sequence. It comprises two α-helices connected by a relatively poorly conserved loop. This structure is essential for the formation of homo- or hetero-dimers by *bHLH* transcription factors [3, 5, 6]. The basic domain is located at the N-terminus of the conserved *bHLH* domain; its DNA-binding capacity depends on the key amino acid residues in the basic region and the number of basic amino acid residues in the basic region [5–7]. Pires and Dolan [8] used the entire genome sequence of nine terrestrial plants and algae to classify the evolutionary relationship of *bHLH* transcription factors in plants. The *bHLH* transcription factors of these different plants are divided into 26 subfamilies. Twenty of these subfamilies are present in the common ancestor of existing mosses and vascular plants, and six subfamilies continue to differentiate in vascular plants. Many *bHLH* genes have been identified in the plant kingdom; for example, in model plants, 147 *bHLH* transcription factors in *Arabidopsis* were divided into 21 subgroups [9] and 167 *bHLH* transcription factors of rice were divided into 22 subgroups [10]. The *bHLH* family has been widely identified in many plants, including *Brassica rapa* ssp. *pekinensis* [11], *Solanum lycopersicum* [12], common bean [13], *Malus pumila* [14], *Arachis hypogaea* [15], *Brachypodium distachyon* [16], *Zea mays* [17], *Triticum aestivum* [18], *Phyllostachys edulis* [19], *Carthamus tinctorius* [20], Chinese jujube [21], *Piper nigrum* [22], Jilin ginseng [23], *Ananas comosus* [24], *Fagopyrum tataricum* [25], and sorghum [26]. The *bHLH* genes have been conserved in the evolutionary history of the plant kingdom. The expansion of this family is closely related to plant evolution and diversity [1, 8] in higher plants as well as in lower plants or non-plants, such as algae, mycobacteria, lichens, and mosses [1]. *bHLH* genes are mainly involved in the defence response to drought, heat, cold and high salt stresses unique to the terrestrial environment. Therefore, whole-genome analysis of the *bHLH* family in different species will help to understand the evolution of organisms, such as green algae, to adapt to environmental changes, along with the evolutionary origin of flowering plants.

Plant *bHLH* transcription factors can have biological roles as both transcriptional activators and repressors, positive and negative regulatory roles in the physiological and biochemical processes of plant light signal transduction, and influence on the development of plant tissues and organs. *SPATULA* is the first gene found in *Arabidopsis thaliana* that affects the formation of floral organs and encodes bHLH proteins. Studies on *SPATULA* mutants have shown that it can promote the growth of plant carpel edges and internal pollen tissues [27]. Heisler et al. proposed that *SPATULA* controls the development of specific tissues of shoot apex meristems, leaves, petals, stamens, and roots [28]. *UDT1* encodes a bHLH protein, isolated from rice [29]. *UDT1* significantly affects the differentiation and microspore formation of anther wall and pollen mother cells at the meiotic stage, thereby regulating the development of rice stamens. *AtSPCH* plays an important regulatory role in the development of *Arabidopsis* stomata [30]. The *bHLH* gene *Tb1* cloned from maize by Doebley et al. controls the growth and development of buds, side branches, and male flowers in the leaf axils of maize [31]. The *bHLH* transcription factor family has been shown to play a vital role in plant resistance to harsh environmental factors, such as drought resistance, salt tolerance, and cold tolerance. For example, *ICE1* and *ICE2* in *Arabidopsis* and their homologous genes in other species can respond to cold stress response processes [32–34]. Overexpression of *AtbHLH*92 gene in *Arabidopsis* can significantly enhance the tolerance of plant to salt damage and osmotic stress [35]. In *Populus euphratica*, the drought resistance of plants overexpressing *PebHLH*35 was higher than that of the wild type [36]. *TabHLH39* has different expression levels in wheat roots, stems, leaves, glumes, pistils, and stamens, and verexpression of *TabHLH39* can enhance the tolerance of *Arabidopsis* plants to drought, salt, and freezing stress in seedlings [37]. *FtbHLH3* is a *bHLH* gene isolated from tartary buckwheat that can be expressed upon exposure to polyethylene glycol (PEG) and abscisic acid (ABA) [38].

Foxtail millet (*Setaria italica* L.) is an important food and feed crop. It is one of the oldest cultivated millet crops and is widely cultivated in arid and semi-arid regions in Asia and Africa [39]. It is a diploid C4 panicle crop with a small genome, short life cycle, strong resistance to stress, and a highly conserved genome structure relative to its ancestor, Corgi [40, 41]. Millet has been used as a model monocot crop for abiotic stress resistance research [42]. Although the *bHLH* gene family may play important roles in abiotic stress resistance and plant development, this family has not been identified in millet. The main gene families identified in this plant are *WD40* [43], *MYB* [44], *AP2/ERF* [45], *ALDH* [46], *Dof* [47], *SOD* [48], *HD-Zip* [49], *SSPs* [50], *CDPK* [51], and *LIM* [52], among others. The genomic sequence of foxtail millet has been reported, laying a foundation for studying the verify, evolution, and expression of genome-wide *SibHLH* genes [40, 41]. In this study, we identified 187 *bHLH* genes and divided them into 21 major groups and two orphan genes. In addition, the exon-intron structure, the motif compositions, chromosomal spread, gene duplications, and evolutionary relationships were analysed. The expression of *SibHLH* members under different tissues and abiotic stresses is discussed. These findings provide valuable clues for future functional identification and evolutionary relationship studies of foxtail millet.

## Results

### Identification of *bHLH* genes in *S. italica*

To identify all *bHLH* genes in the *S. italica* genome, two BLAST methods were used to identify all possible *bHLH* members (Additional file 1: Table S1). They were then renamed *SibHLH1* to *SibHLH187* according to their location on the *S. italica* chromosomes. The basic characteristics that were analysed included molecular weight (MW), isoelectric point (pI), coding sequence length (CDS), and subcellular localization (http://cello.life. nctu.edu.tw/).

Of the 187 SibHLH proteins, SibHLH111 was the smallest with 79 amino acids. The largest was SibHLH150 with 897 amino acids. The molecular weight of the proteins ranged from 9.03 kDa (SibHLH147) to 97.04 kDa (SibHLH150). The pI ranged from 4.56 (*SibHLH6* and *SibHLH68*) to 12.03 (*SibHLH*46), with a mean of 6.88. Of all the *SibHLH* genes, five contained the *bHLH*-*MYC* domain. In the predicted subcellular localisation results, 150 *SibHLHs* were located in the nucleus, 16 in the chloroplast, 12 in the cytoplasm, seven in the mitochondria, one (*SibHLH23*) in the endoplasmic reticulum (ER), and one (*SibHLH14*) in the peroxisome (Table S1). The ratio of *SibHLH* genes to total genes in the *S. italica* genome was approximately 0.48%, which was less than that in *Arabidopsis* (0.59%) [9] and buckwheat (0.49%) [25], but higher than that in rice (0.44%) [10], poplar (0.40%) [1], and tomato (0.46%) [12].

### Multiple sequence alignment, phylogenetic analysis, and classification of *SibHLH* genes

**Fig. 1:**
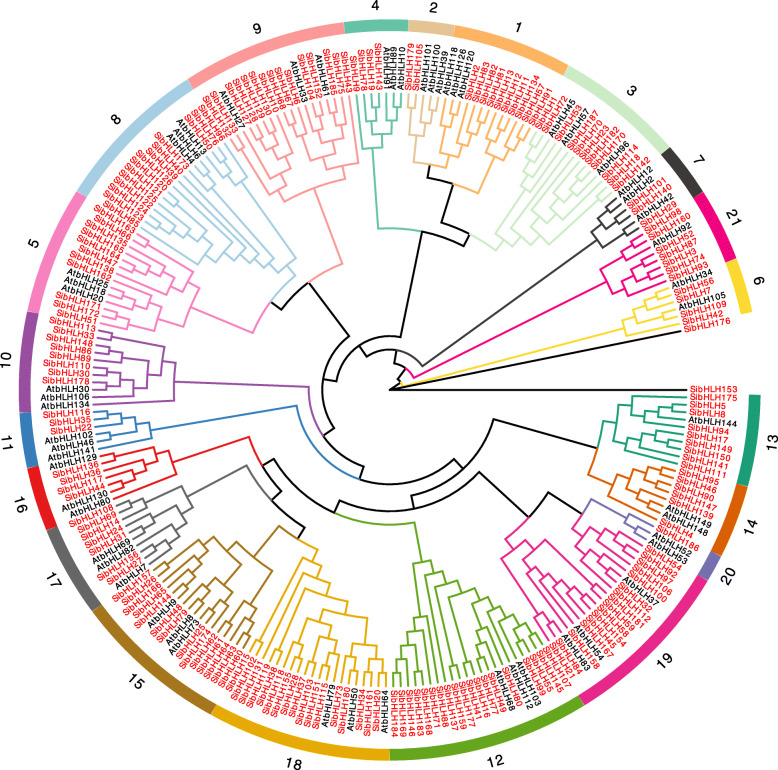
Unrooted phylogenetic tree showing relationships among *bHLH* domains of *S. italica* and *Arabidopsis*. The phylogenetic tree was derived using the neighbour-joining method in MEGA7.0. The tree shows the 21 phylogenetic subfamilies and one unclassified group (UC), which are denoted in red font. *bHLH* proteins from *Arabidopsis* are denoted by the prefix ‘At’

To investigate the phylogenetic relationship of the foxtail millet bHLH proteins, we constructed a phylogenetic tree of foxtail millet (187 SibHLHs) and *Arabidopsis thaliana* (56 AtbHLHs) using the neighbour-joining method (Fig. 1; Additional file 1: Table S1). According to the previously proposed classification method and topological structure [8, 9], 243 bHLH proteins in the phylogenetic tree were divided into 21 main clades (groups 1–21). The unclassified group (UC) contained two *SibHLH* genes (*SibHLH153* and *SibHLH176*). One hundred and eighty-five SibHLH proteins were distributed unevenly in 21 subfamilies, consistent with the classification of bHLH proteins in Arabidopsis [9, 10]. These data indicate that these proteins have been maintained during the long-term evolution of *S. italica*. Two bHLH orphan proteins formed two different topological structures from AtbHLH proteins, which may be a new characteristics in the evolution of *S. italica* diversity (Fig. 1, Table S1).

Among the 21 subfamilies, subfamily 12 displayed the largest number of members (19 *SibHLH*s) and subfamily 20 displayed the fewest (one SibHLH protein). The phylogenetic tree results showed that some SibHLHs clustered tightly with AtbHLHs (bootstrap support ≥70), suggesting that these proteins may be orthologous and have similar functions.

**Fig. 2:**
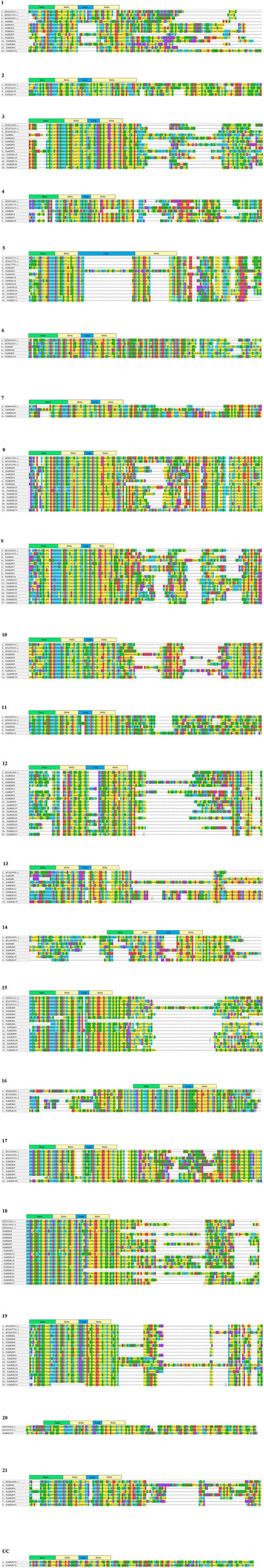
Multiple sequence alignment of the *bHLH* domains of the members of 21 phylogenetic subfamilies and one unclassified group (UC) of the *SibHLH* protein family. The scheme at the top depicts the locations and boundaries of the basic, helix, and loop regions in the *bHLH* domain

The AtbHLH proteins and those from subgroups 1 to 21 were randomly selected as representative groups for further multiple sequence comparisons (Fig. 2, Table S1). The basic region was considered 17 amino acids based on previous study [9]. The bHLH domains of *S. italica* span approximately 50–60 amino acids, which is different from *Arabidopsis* [9] and rice [10]. As shown in Fig. 2, although the characteristic bHLH domain is well conserved in *S. italica*, it is differentiated and diversified in many SibHLH proteins [7, 10, 53]. The loop region was the most divergent region in terms of amino acid domain, especially in subfamily 1, 10, 12, and 14, which has been observed in other plant bHLH proteins, including Arabidopsis [9], *Solanum lycopersic* [12], and *Fagopyum tataricum* [25].

### Conserved motifs and gene structure analysis of *SibHLH* genes

**Fig. 3:**
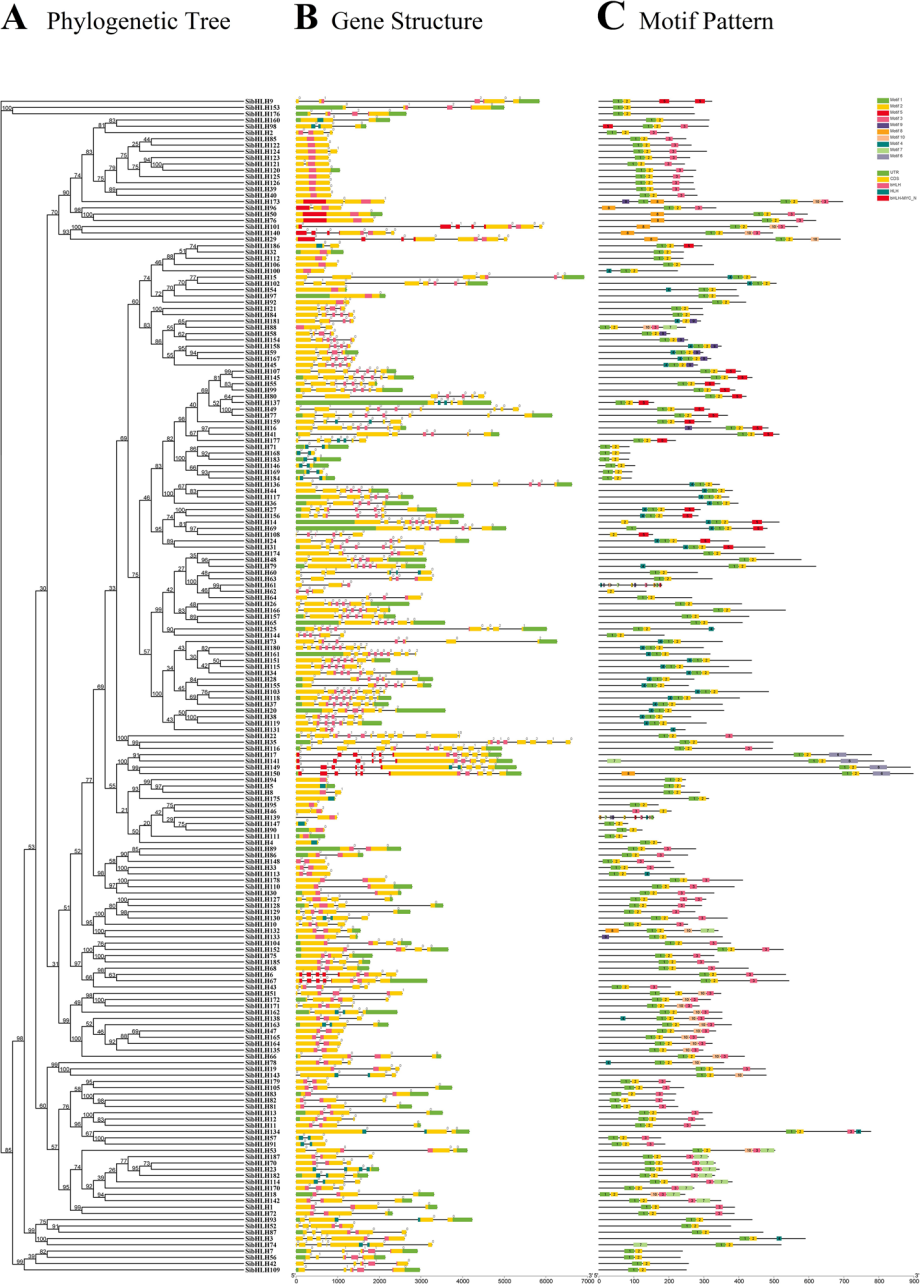
Phylogenetic relationships, gene structure analysis, and motif distributions of *S. italica bHLH* genes. **A** Phylogenetic tree constructed using the neighbour-joining method with 1000 replicates for each node. **B** Exons and introns are indicated by yellow rectangles and grey lines, respectively. **C** Amino acid motifs in the *SibHLH* proteins (1–10) are represented by coloured boxes. The black lines indicate relative protein lengths

By comparing the genomic DNA sequences of *SibHLH* genes, we obtained the intron and exon structure of *SibHLH* genes to further understand the structural composition of these genes (Fig. 3, Additional files 1 and 2: Tables S1 and S2). A comparison of localization and number of the exon-intron structures revealed that the 187 *SibHLH* genes had different numbers of exons, varying from 1 to 13 (Fig. 3A, B). In addition, 24 (12.83%) genes contained one exon, and the remaining genes had two or more exons. The 24 genes lacking introns belonged to four subfamilies (8, 13, 14, and 19), but mainly to subfamily 8. The greatest proportion of *SibHLH* genes (*n* = 34) had two exons. Group 11 had the most exons, with 11 (*SibHLH35* and *SibHLH116*) or 13 exons (*SibHLH22*). Groups 8, 10, 14, and 20 contained one or two exons. Further analyses indicated that groups 12, 15, and 21 showed more diversity in intron numbers. In general, the gene structures in the same subfamily exhibited similarities, although the locations of the exons were different.

The motifs of 187 SibHLH proteins were analysed online using MEME software to further study the characteristic regions of SibHLH proteins (Fig. 3C, Table S2). Ten conserved motifs were found in SibHLH proteins (Fig. 3C). As shown in Fig. 3C, the motifs 1 and 2 were widely distributed in the *SibHLH*s, except in *SibHLH46*, *SibHLH62*, *SibHLH108*, *SibHLH131,* and *SibHLH187*. The two motifs were very close to each other in SibHLH proteins. *SibHLH* members within the same group generally share a similar motif composition. For example, groups 4, 6, 8, 10, 11, 12, 13, 14, 19, and 21 members contained motifs 1 and 2; groups 1, 2, and 3 contained motifs 1, 2, and 3; group 5 contained motifs 1, 2, 10, and 3; group 20 contained motifs 1, 2, and 5; group 16 and 18 contained motifs 4, 1, and 2; group 17 contained motifs 4, 1, 2, and 5; and group 7 contained motifs 4, 1, 2, and 10. Some motifs were present only in specific subfamilies. In addition, motif 7 was specific to groups 3, 12, 13, and 21, whereas motif 8 was specific to groups 7, 8, 9, and 13. Further analysis showed that some motifs could only be distributed in specific locations of the pattern. Motifs 1 and 2 were always distributed at the start of the pattern in groups 1, 2, 3, 6, 10, 11, 14, and 20. Motif 4 was almost always distributed at the start of groups 16 and 18. Motif 3 was almost always distributed at the end of the pattern in groups 2 and 5. Motif 5 was distributed at the end of the pattern in group 17. The similarity of motif composition of the same subfamily indicates the conservation of the protein structure of the subfamily. The functions of these conserved motifs remain to be elucidated. Overall, the conserved motif composition and gene structure of the same subfamilies were similar, supporting the phylogenetic tree population classification.

### Chromosomal spread and gene duplication of *SibHLH* genes

**Fig. 4:**
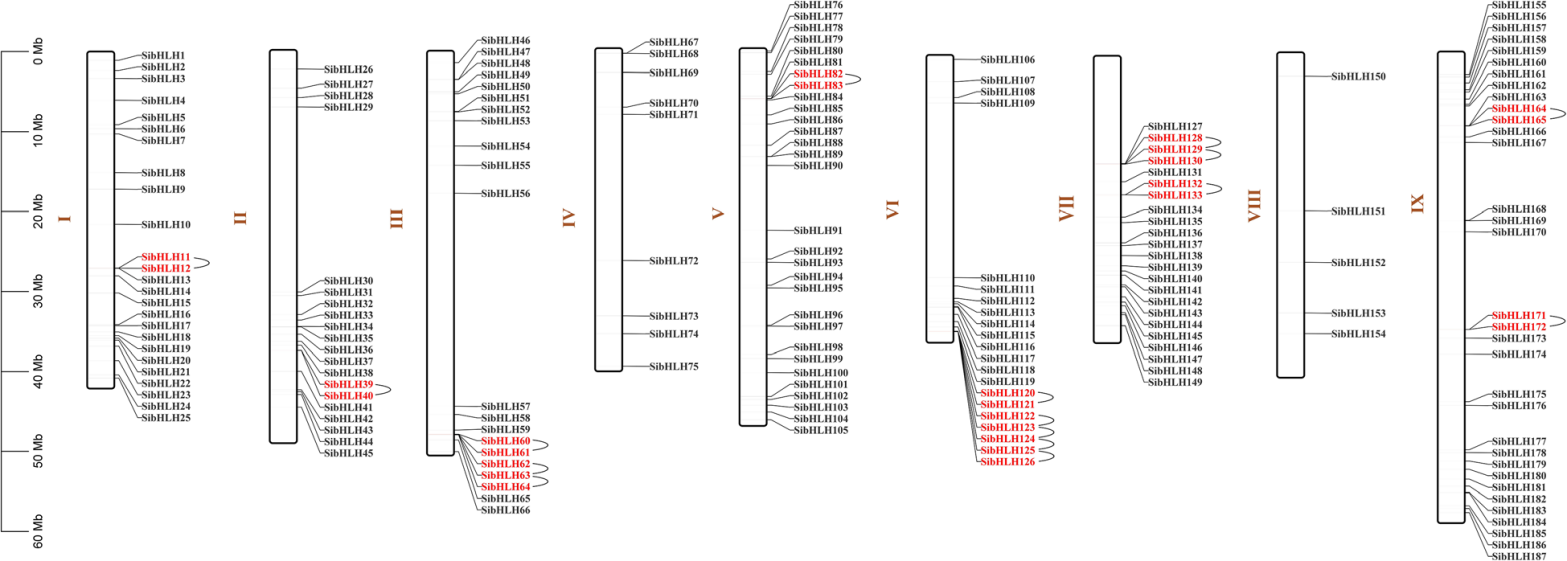
Schematic representation of the chromosomal distribution of the *S. italica bHLH* genes. Vertical bars represent the chromosomes of *S. italica*. The chromosome number is indicated to the left of each chromosome. The scale on the left represents chromosome length

The distribution of the 187 *SibHLH* genes on chromosomes (Chr) 1 (I) to 9 (IX) was uneven (Fig. 4, Additional file 3: Table S3). Each *SibHLH* name corresponds to its physical position from the top to the bottom of *S. italica* Chr1 to Chr9. Chr9 contained the largest number of *SibHLH* genes (33 genes, ~ 17.65%), followed by Chr5 (30 genes, ~ 16.04%). Chr8 contained the lowest (5 genes, ~ 2.67%). Chr3 and Chr6 each contained 21 (~ 11.23%) *SibHLH* genes. Chr1, Chr2, Chr4, and Chr7 contained 25 (~ 13.37%), 20 (~ 10.70%), 9 (~ 4.81%) and 23 (~ 12.30%) *SibHLH* genes, respectively. Many *bHLH* gene duplication events were evident in *S. italica*. Two or more identical genomic regions were found within a 200 kb chromosomal region, which is defined as a tandem repeat event [54]. Sixteen tandem duplication events involving 27 *SibHLH* genes were observed on chromosomes 1, 2, 3, 5, 6, 7, and 9 (Fig. 4). *SibHLH63, SibHLH123, SibHLH124, SibHLH125,* and *SibHLH129* each had two tandem repeat events (*SibHLH63* and *SibHLH62* / *SibHLH64*; *SibHLH123* and *SibHLH122* / *SibHLH124*; *SibHLH124* and *SibHLH123* / *SibHLH125*; *SibHLH125* and *SibHLH124* / *SibHLH126*; *SibHLH129* and *SibHLH128* / *SibHLH130*). All *SibHLH* genes that formed tandem repeat events belonged to the same subfamily. For example, *SibHLH164* and *SibHLH165* were tandem repeat genes that were clustered together in subfamily 5 (Fig. 4, Table S3).

**Fig. 5:**
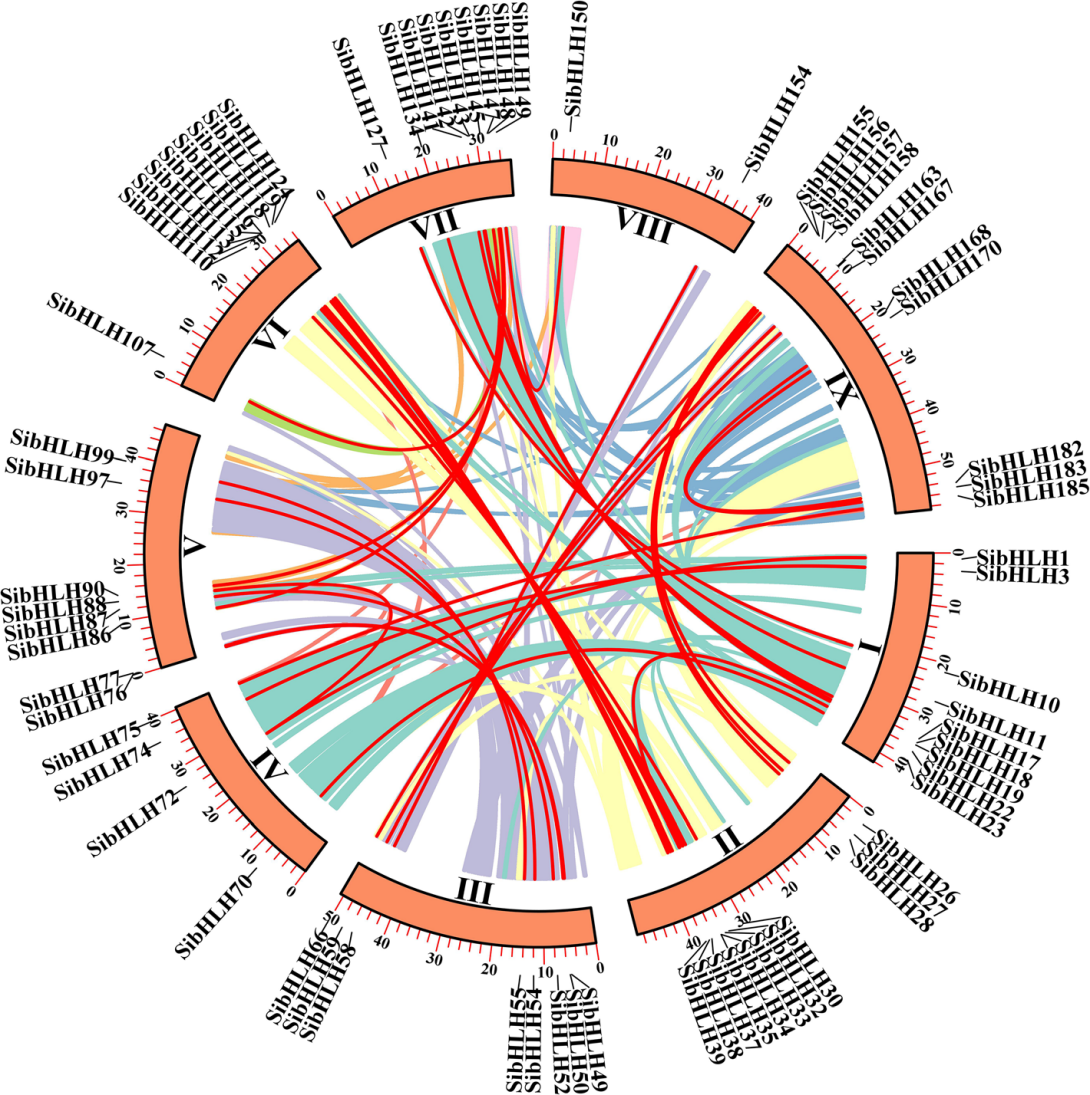
Schematic representation of the chromosomal distribution and interchromosomal relationships of *S. italica bHLH* genes. Coloured lines indicate all synteny blocks in the *S. italica* genome, and the red lines indicate duplicated *bHLH* gene pairs. Chromosome number is indicated at the bottom of each chromosome

There were 38 pairs of segmental duplications in *SibHLH* genes (Fig. 5, Additional file 4: Table S4). As shown in Figs. 5, 73 (39.04%) paralogs were identified in the *SibHLH* genes, indicating an evolutionary relationship in these *SibHLH* genes. LG2 had the most *SibHLH* menmbers (*n* = 13) and LG8 had the least (*n* = 2). As expected, most genes were linked within their subfamily, except for *SibHLH72* and *SibHLH88*. For all identified *SibHLH* genes, groups 3 and 19 had the largest number of linked genes (5 *SibHLH* genes). In addition, groups 12 and 18 had four genes, while groups 1, 4, 5, 14, 15, 16, and 17 had only one *SibHLH* gene (Table S4). These results indicate that some *SibHLH* genes may have been produced by gene duplication and that these tandem duplication events played a major role in the occurrence of new functions in *S. italica* evolution, along with the amplification of the *SibHLH* gene family.

### Synteny analysis of *SibHLH* genes

**Fig. 6:**
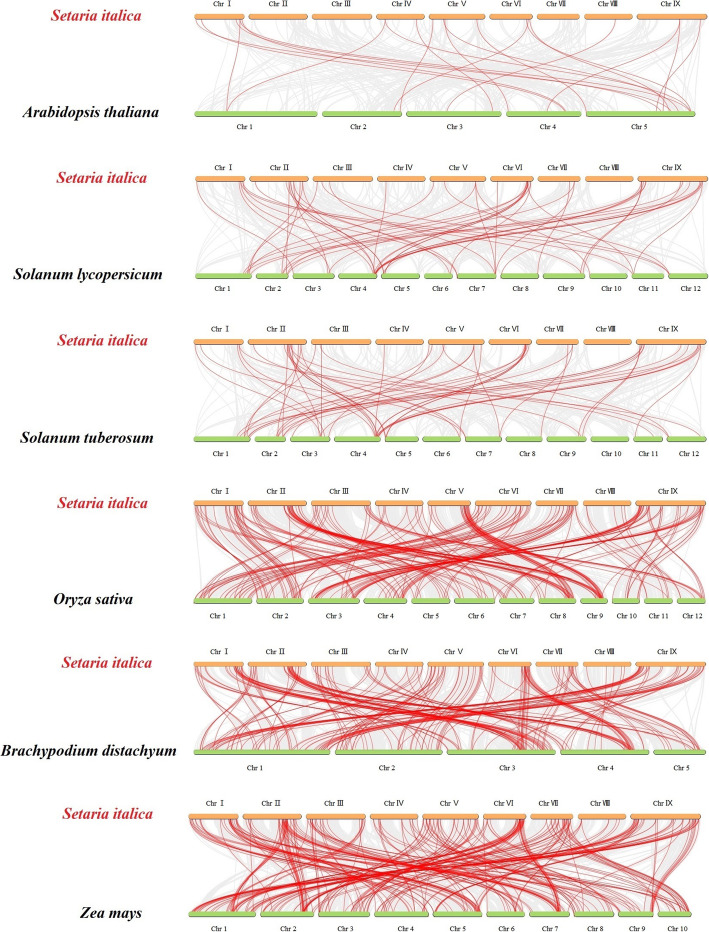
Synteny analyses of the *bHLH* genes between *S. italica* and six representative plant species (*Arabidopsis thaliana*, *Solanum lycopersicum*, *Solanum tuberosum, Brachypodium distachyon*, *Oryza sativa* subsp. *indica*, and *Zea mays*). Gray lines on the background indicate the collinear blocks in *S. italica* and other plant genomes; red lines highlight the syntenic *S. italica bHLH* gene pairs

To further elucidate the evolutionary relationship of bHLH proteins in several plants, six comparative synteny maps of *S. italica* associations with six representative species were constructed. These species included three dicotyledons (*A. thaliana*, *S. lycopersicum*, and *S. tuberosum*) and three monocotyledons (*B. distachyon*, *O. sativa*, and *Zea mays*) (Fig. 6, Additional file 5: Table S5). A total of 153 *SibHLH* genes displayed syntenic relationships with those in *A. thaliana* (*n* = 17), *S. lycopersicum* (*n* = 39), *S. tuberosum* (*n* = 44), *O. sativa* (*n* = 137), *B. distachyon* (n = 137), and *Z. mays* (*n* = 145) (Table S5). The number of orthologous pairs between the other six species (*A. thaliana*, *S. lycopersicum*, *S. tuberosum*, *O. sativa*, *B. distachyon*, and *Z. mays*) were 20, 53, 53, 211, 196, and 268, respectively. Some *SibHLH* genes were associated with at least three syntenic gene pairs (particularly between *S. italica* and *Z. mays bHLH*). These genes included *SibHLH32, SibHLH37, SibHLH38, SibHLH107, SibHLH112, SibHLH119, SibHLH149*, and *SibHLH150*, suggesting their important roles during evolution.

In addition, many collinear gene pairs (with 99 *SibHLH* genes) identified between *S. italica* and *B. distachyon*, *O. sativa*, and *Z. mays* were not identified in *S. italica*, *A. thaliana*, *S. tuberosum*, and *S. lycopersicum*. These included *SibHLH2* with BGIOSGA006833 / KQJ94140 / Zm00001d016257 and *SibHLH6* with BGIOSGA007229 / PNT65750 / Zm00001d053895. Similar patterns were also found among *S. italica* with *O. sativa/ B. distachyon* / *Z. mays*, which indicated that these paralogous genes may be gradually formed after the independent differentiation of monocotyledons (Table S5). In addition, some *SibHLH* genes were associated with at least one paralogous pair among in six plants (especially between *S. italica* and *Z. mays*). These genes included *SibHLH1, SibHLH23, SibHLH79, SibHLH87, SibHLH114, SibHLH117, SibHLH11,* and *SibHLH181*, suggesting that these homologous genes already existed before the ancestral divergence. To better observe the evolutionary constraints of the 187 *SibHLH* genes, the *SibHLH* genes were subjected to the Tajima D neutrality test. The calculated D of 7.05 deviated markedly from 0, suggesting that the *SibHLH* gene family might have been involved in the purification and selection pressure during evolution process.

### Evolutionary analysis of *SibHLH* and *bHLH* genes of several different species

**Fig. 7:**
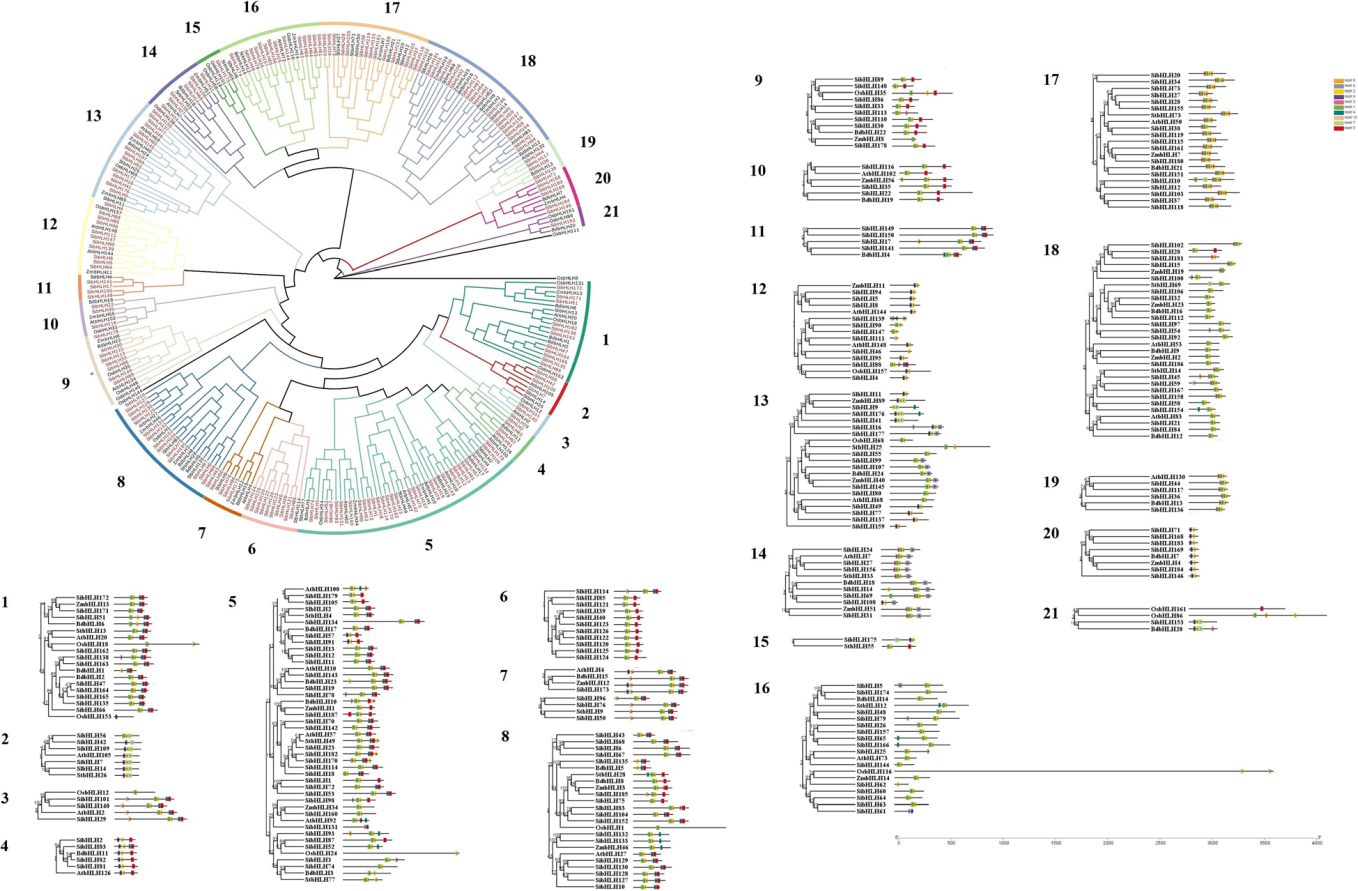
Phylogenetic relationship and motif composition of the *bHLH* proteins from *S. italica* with six different plant species (*Arabidopsis thaliana*, *Solanum lycopersicum*, *Solanum tuberosum, Brachypodium distachyon*, *Oryza sativa* subsp. *indica*, and *Zea mays*) Outer panel: an unrooted phylogenetic tree constructed using Geneious R11 with the neighbour-joining method. Inner panel: distribution of conserved motifs in *bHLH* proteins. The differently coloured boxes represent different motifs and their positions in each *bHLH* protein sequence. The sequence information for each motif is provided in Additional file 2 (Table S2).

To analyse the evolutionary relationship of the trihelix family of bHLH proteins in *S. italica* and six other plants (*A. thaliana*, *S. tuberosum*, *S. lycopersicum*, *O. sativa*, *B. distachyon*, and *Z. mays*), an unrooted NJ tree was constructed. The tree contained ten conserved motifs according to the MEME web server relative to the protein sequences of 298 bHLH proteins (Fig. 7, Additional file 2: Table S2). The detailed genetic correspondence was shown in Additional files 1 and 2 . These bHLH proteins in the phylogenetic tree were divided into 21 clades (groups 1–-21). Except for a few SibHLH proteins, such as SibHLH42, SibHLH61*,* and SibHLH131, all other SibHLH proteins contained motif 2. In addition, many motifs existed only in a few specific *SibHLH* branches, such as the motifs 8 and 10. In general, the bHLH proteins of *O. sativa*, *Z. mays,* and *S. italica* on the same branch had similar motif compositions. Similar motifs compositions tend to be distributed in some specific bHLH protein subfamilies. For example, serial motifs 10, 1, 2, 4, and 5 tended to gather within the group 7, and serial motifs 3, 1, 2, and 8 tended to gather within group 17.

### Expression patterns of *SibHLH*s in several plant organs

**Fig. 8:**
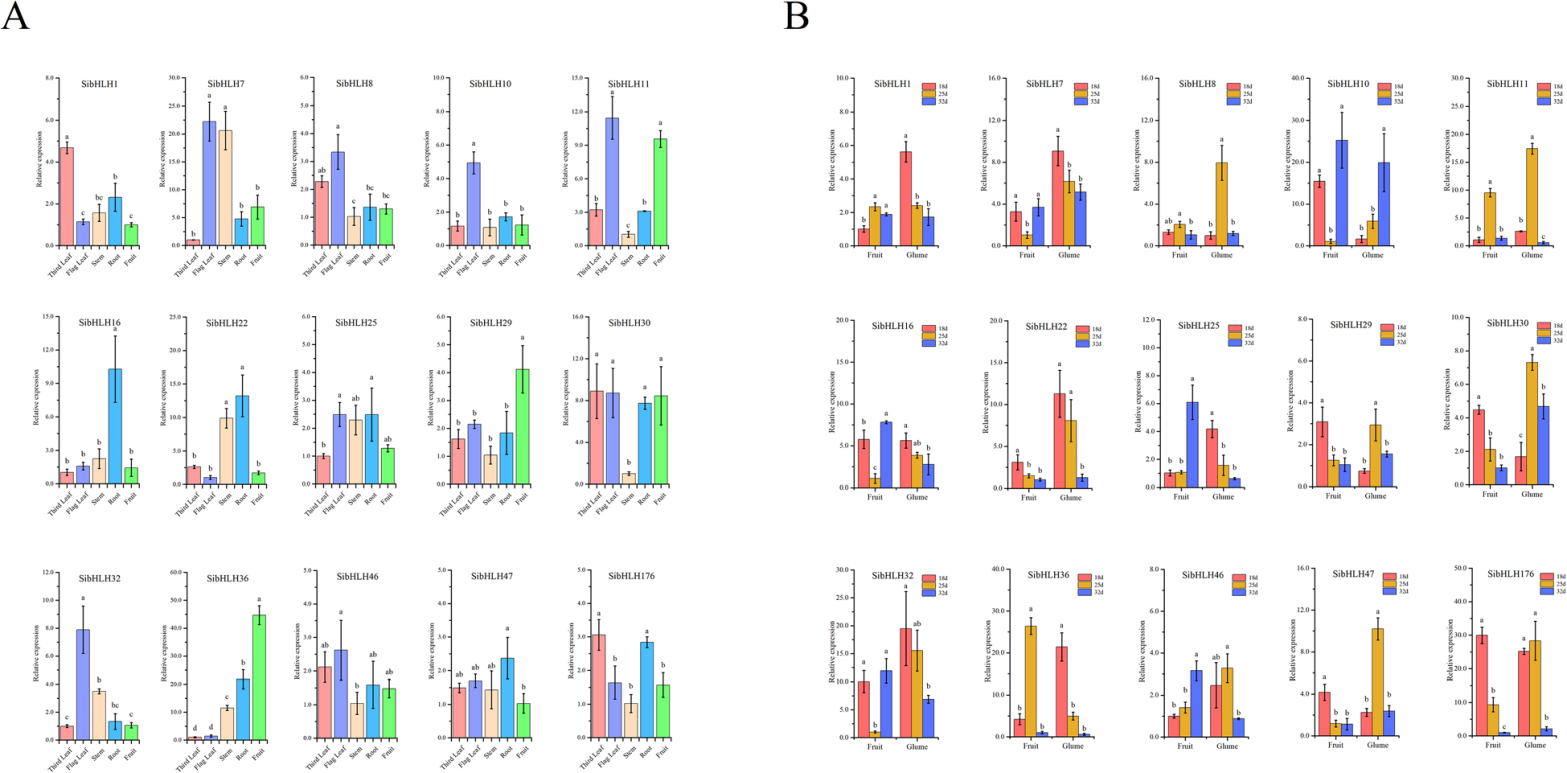
Expression patterns of 15 *S. italica bHLH* genes in several plant organs. **A** Expression patterns of 15 *S. italica bHLH* genes in the third leaf, flag leaf, root, stem, and fruit organs were examined via qRT-PCR. Error bars are obtained from three measurements. The SE is selected as the value of bar. The same below. **B** Expression patterns of 15 *S. italica bHLH* genes were examined during different fruit development stages: 18 DPA (early filling stage), 25 DPA (middle filling stage), and 32 DPA (initial maturity stage). Lowercase letters above the bars indicate significant differences (α = 0.05, LSD) among the treatments

To investigate the potential roles of the *SibHLH* genes, real-time PCR was used to detect the expression of 15 individual members from different subfamilies. As far as possible, these subfamilies have distant clustering relationships and significant differences in their amino acid structures. The accumulation of the transcriptional products of 15 *SibHLH* genes from different subfamilies in five organs (third leaf, flag leaves, stems, roots, and fruits) was evaluated (Fig. 8A). Some genes were preferentially expressed in some tissues of *S. italica*. Most genes were expressed in all the organs. Four genes (*SibHLH7*, *SibHLH8*, *SibHLH10,* and *SibHLH32*) displayed highest expression in the flag leaves; the expression of two genes (*SibHLH29* and *SibHLH36*) was highest in the fruits, whereas the expression of *SibHLH16* and *SibHLH22* was highest in the roots, and *SibHLH7* and *SibHLH22* were highly expressed in the stem.

Some *SibHLH*s may regulate the fruit development of *S. italica*, thus affecting its nutritional composition and the development rate. This was assessed by studying the expression of 15 *SibHLH* genes at 18 (early filling stage), 25 (middle filling stage), and 32 (initial maturity stage) days post-anthesis (DPA) to identify the genes that may regulate the development of fruits of *S. italica* [25]. The expression levels of most *SibHLH* genes were different in fruits and glumes during the three fruit development stages. In the fruits of foxtail millet, the expression of three genes (*SibHLH1, SibHLH25,* and *SibHLH46*) increased with fruit development, whereas the expression levels of five *SibHLH* genes (*SibHLH22, SibHLH29, SibHLH30, SibHLH47,* and *SibHLH176*) decreased with fruit development. The expression levels of two genes (*SibHLH11* and *SibHLH36*) were highest at DPA 25, whereas the expression levels of four genes (*SibHLH7, SibHLH10, SibHLH16,* and *SibHLH32*) were lowest at 18 DPA (Fig. 8B). The expression levels of most genes (*SibHLH1, SibHLH7, SibHLH16, SibHLH22, SibHLH25, SibHLH32, SibHLH36, SibHLH46,* and *SibHLH176*) decreased with fruit development, whereas the expression of *SibHLH10* increased.

The expression patterns of *SibHLH* members have shown many coordinated expressions in several plant organs (Fig. S1). Most *bHLH* genes showed significant positive correlations; for example, five genes *SibHLH8*, *SibHLH32*, *SibHLH46*, *SibHLH47,* and *SibHLH176* were significantly positively correlated, and *SibHLH11*, *SibHLH29,* and *SibHLH30* were significantly positively correlated. On the other hand, some pairs of *SibHLH* genes (*SibHLH25* and *SibHLH29* / *SibHLH30*; *SibHLH10* and *SibHLH36*; *SibHLH1* and *SibHLH29*) were significantly negatively correlated.

### Expression patterns of *SibHLH* genes in response to different treatments

**Fig. 9:**
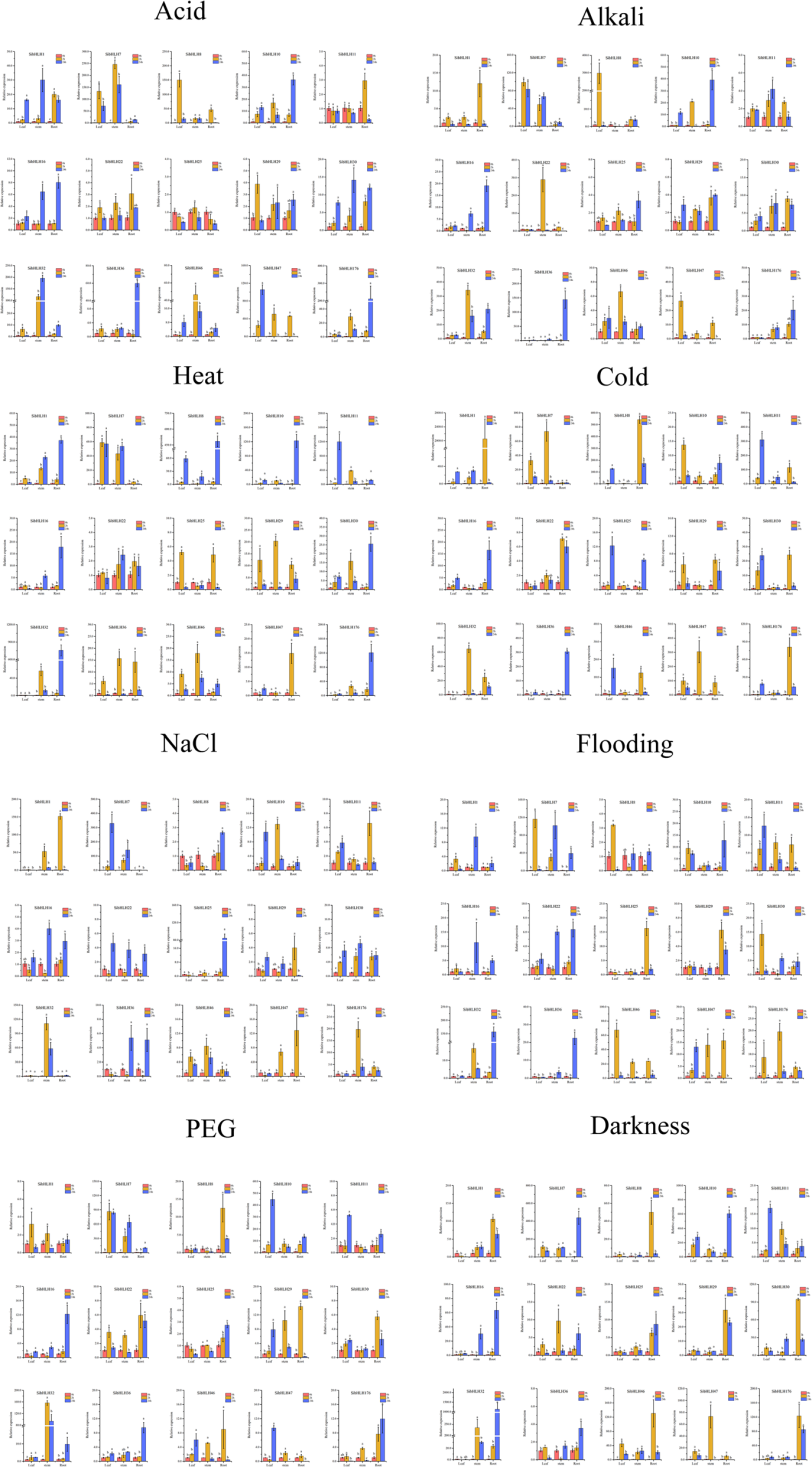
Gene expression of 15 *S. italica bHLH* genes in plants subjected to abiotic stresses (acid, alkali, PEG, NaCl, heat, cold, flooding, and dark) at the seedling stage. The expression patterns of 15 *S. italica bHLH* genes in leaf, root, and stem organs were examined via qRT-PCR. Error bars were obtained from three measurements. Lowercase letters above the bars indicate significant differences (α = 0.05, LSD) among the treatments

To further determine whether the expression of *SibHLH* genes was influenced by different abiotic stresses, the expression of 15 *SibHLH* members was examined under eight abiotic stresses: acid (0.1 M), alkali (0.2 M), PEG (10%), NaCl (5%), heat (40 °C), cold (4 °C), flooding, and darkness. qRT-PCR analysis was performed to analyse the expression patterns of the 15 *SibHLH* genes in the roots, leaves, and stems to response the different abiotic stresses (Fig. 9). Some *SibHLH* genes were significantly upregulated or inhibited under different stresses. Interestingly, the expression levels of *SibHLH*s showed changes over time or in different organs, depending on the specific treatments. For example, under heat stress, *SibHLH29* and *SibHLH36* were significantly upregulated and then downregulated. *SibHLH16* expression was significantly upregulated in the roots at 24 h, whereas it was significantly downregulated in the leaves. Under acid stress, *SibHLH25* was significantly downregulated in the roots and leaves, but *SibHLH16* was significantly upregulated. Several genes showed opposing expression patterns during different treatments. Transcript levels of many *SibHLH* genes, such as *SibHLH16* was upregulated in stems and downregulated in leaves by heat stress treatment, whereas its expression pattern was reversed by cold stress. The expression of some genes showed similar patterns under different stress treatments. For example, *SibHLH16* expression was unchanged first and then significantly upregulated in the roots by the different treatments. Other genes showed changes in specific organs. For instance, *SibHLH7* responded significantly to acid and alkali treatment in the leaves and stems, and *SibHLH22* responded significantly to cold treatment in the roots. Furthermore, correlations between *SibHLH* gene expression patterns were observed (Fig. S2). Negative correlations were observed among the most *SibHLH* genes. However, a few *SibHLH* genes were significantly positively correlated, such as *SibHLH29* with *SibHLH30* / *SibHLH46*, as well as *SibHLH10* with *SibHLH16* / *SibHLH32* (*P* < 0.05).

## Discussion

### *SibHLH* gene structure and evolutionary analyses

The encoded proteins of 187 *SibHLH* genes were structurally distinct, suggesting the complexity of their possible functions (Figs. 1 and 2, Additional file 1: Table S1). The DNA-binding properties were determined by the conserved amino acids in the DNA-binding domain of transcription factors. Similar transcription factors were bound to the same cis-acting elements. The DNA-binding ability of the basic region of *SibHLH*s was analysed [4, 9] (Table S1). Atchley et al. [55] identified that Glu-9 and Arg-13 in the basic region are essential amino acid residues for binding to the E- and G-box in the H / K5-E9-R13 mode. A total of 132 *SibHLH*s (70.6%) were classified as E-box binding proteins, 95 *SibHLH*s (50.8%) were classified as G-box binding proteins, and 37 *SibHLH*s (19.8%) were classified as non-G-box binding proteins. In addition, there were 30 non-E-box-binding genes (16.0%). The remaining 25 members (13.4%) were not considered capable of binding to DNA because of a lack of Glu-13 or Arg-16 in the alkaline region (Fig. 2, Attachment 1: Table S1). The highest proportion of *SibHLH* was G-box binding protein, but the value was lower than that of *O. sativa* (*n* = 95, 56.9%) and *A. thaliana* (*n* = 89, 60.5%) [10]. Previous studies have found that some key amino acid residues can bind DNA, *bHLHs*, and other transcription factors to form homo- or hetero-dimers, which can change the interaction between molecules to generate new DNA-binding sites and further generate new functions [9, 56]. For example, His-5, Glu-9, and Arg-13 are closely related to DNA-binding activity, whereas Leu-27 and Leu-57 determine whether *bHLH* transcription factors can form dimers, which is related to the regulatory function of the bHLH protein. There are two connected helical structures in most SibHLH genes composed of hydrophobic amino acids, including leucine at positions 23 and 64 (Leu23 / 64), leucine or isoleucine at position 54 (Leu / Iso54), and valine (Val61) at position 61. Leu23 and Leu52 residues in the HLH region are necessary for dimer formation [1, 57]. In the present study, the retention rates of Leu-25 and Leu-57 of *SibHLH*s were 97 and 91%, respectively (Fig. 2), which were lower than those of *S. lycopersicum* (99 and 97%, respectively) [11] and *Citrus reticulata* (both 100%) [58]. Notably, all SibHLH proteins of subfamily 1 lacked Leu-57. We also observed this in sorghum, where none of the proteins had the ability to form dimers in subfamily 1 [26]. However, whether this is related to the independent differentiation of C4 plants requires further investigation.

A total of 185 *SibHLH* genes were identified and classified into 21 subfamilies according to their phylogenetic relationships with known *bHLH* genes from *Arabidopsis* (Fig. 1) [9, 10]. These results indicate their indispensable role in the evolution and development of *S. italica*. Compared with *A. thaliana*, these subfamily genes were not lost in *S. italica*, despite its status as a monocotyledonous C4 plant, suggesting that the diversity of most of the bHLH proteins may have been established in early land plants. In addition, further differentiation of *bHLH* genes in monocotyledonous plants was supported by the presence of two orphan genes (*SibHLH153* and *SibHLH176*). Our findings support the view that the *bHLH* family of plants evolved in a highly monophyletic manner [8], rather than the absence of several types of parallel evolution. Based on the evolutionary tree, the number of members was greater in *S. italica* group 15 (*n* = 17, 9.8%) and group 18 (*n* = 15, 8.6%), similar to that of *A. thaliana* and rice, suggesting that these *bHLH* gene groups may have experienced stronger expansion in the long evolutionary process under the characteristics of monocotyledons. The number and proportion of members in groups 5, 12, and 21 were significantly higher than those in *Arabidopsis* [9] and similar to those in rice [10]. Whether this differentiation is beneficial for herbaceous and woody plants has not yet been determined.

Significant intron loss or expansion was evident in some *SibHLH* member domains. These may lead to family expansion and the generation of new functions. Genes with few or no introns are generally expressed at low levels in plants [59]. However, genes with a compact structure may facilitate rapid gene expression in response to plant development or abiotic stress [1]. For example, the expression of *SibHLH8* increases rapidly under acid, alkali, and cold stress and may be in response to these abiotic stresses. The extension of gene families and the mechanism of genomic evolution are mainly dependent on tandem repetition and fragment replication [60–63]. We identified 16 tandem repeat events involving 27 *SibHLH* genes (Fig. 4, Table S3), especially on chromosomes 3, 6, and 7. In addition, *SibHLH* genes had 38 pairs of segment duplications (Fig. 5, Table S4). As expected, almost all the expanded genes were mainly within the subfamily, except for *SibHLH*72 (subfamily 3) and *SibHLH*88 (subfamily 12), similar to *A. thaliana* [9], rice [10], and Tartary buckwheat [25]. Therefore, segment duplication of *SibHLH* genes may make a higher contribution to the amplification in the *bHLH* family in foxtail millet. There were many duplication events in *S. italica,* nevertheless,,it was still lower than that of the dicotyledonous plants *Solanum lycopersicum* and *S. tuberosum* [64, 65].

### Expression patterns and function prediction of *SibHLH*s

The pattern of gene expression is an important factor in determining the function and characteristics of *bHLH* genes [25]. In this study, the expression patterns of 15 *SibHLH* genes with significant differences in phylogenetic trees in different organs and different developmental stages of fruits were studied (Fig. 8A, B). Almost all *bHLH*-TFs were significantly differentially expressed (more than 2-fold differences). *SibHLH22* is classified in subfamily 9 and has the highest expression levels in roots and stems. This is similar to the expression pattern of the homologous gene *AtbHLH46*, which regulates the development of roots and stems in *Arabidopsis* [66]. In addition, the expression of *SibHLH8*, *SibHLH10,* and *SibHLH32* in flag leaves of millet was significantly higher than that in the roots, stems, and fruits. Therefore, the possible relationship between these genes and leaf development could be experimentally verified. In addition, *SibHLH8* and *SibHLH11* were highly correlated (Fig. 8A, Fig. S1). *SibHLH8* and *SibHLH11* were highly expressed, not only in the evolutionary relationships in the flag leaves of *S. italica,* but also in fruits and glumes at the middle filling stage (Fig. 8B). The exploration of the evolutionary relationship between *SibHLH* genes and *bHLH*s in other plants revealed a similar evolutionary relationship and function. For example, *SibHLH30* and *AtbHLH30* both belong to subgroup 10 and have similar motif components (Fig. 3, Fig. 7). *SibHLH30* is highly expressed in leaves, including flag leaves and the third leaf. Its expression pattern was similar to that of *AtbHLH30*. Overexpression of *AtbHLH30* can alter auxin balance and vein development in *A. thaliana*, thereby regulating leaf epidermal morphology [67]. *AtbHLH42*, which is classified under subfamily 7, is expressed much more in fruits than in roots [35], similar to the expression pattern of *SibHLH29*. These findings provide a direction for further validation of its function. *AtbHLH18* (*At2g22750*) and *AtbHLH20* (*At2g22770*) were identified in *A. thaliana* and were highly expressed in the roots. They negatively regulate root development and iron uptake by promoting JA-induced FIT protein degradation [68]. *AtbHLH18*, *AtbHLH20,* and *SibHLH47* are closely related, and their tissue-specific expression patterns are similar. Therefore, it is necessary to further validate the regulation of root development by *SibHLH47*. As a typical C4 plant, the development of spikelets and fruits is very important for *S. italica*. Therefore, to identify the *bHLH* genes that may regulate the development of foxtail millet fruits, the expression levels of 15 *bHLH* genes in the pericarp and grain of *S. italica* during the grain-filling stage were investigated in this study. *SibHLH*36 displayed high expression levels in all tissues, with the highest expression observed in fruits. Moreover, *SibHLH*36 expression was the highest in the middle filling stage (green fruit stage). We attempted to further verify the role of *SibHLH*36 in plant growth and fruit development.

To further explore the physiological role of the *bHLH* family in environmental adaptation, we systematically analysed the expression of 15 *SibHLH*s in foxtail millet seedlings under different stressors (Fig. 9). Under drought stress, the expression levels of 13 *SibHLH* genes in the roots, 11 genes in the leaves, and 10 genes in the stems were significantly upregulated. These responses may help foxtail millet adapt to drought conditions. This is consistent with the nature of millet as a drought-tolerant crop. Similar conclusions have been reached for poplar [1] and Tartary buckwheat [25]. *AtbHLH20* (*At2g22770*) is preferentially expressed in root epidermal non-hair cells in subfamily 5 of *A. thaliana* [69]. Similarly, *SibHLH47* responded significantly to eight stresses in roots. Its expression was increased during seven stresses (acid, alkali, NaCl, heat, cold, flooding, and dark) but decreased significantly during PEG stress. *AtbHLH34*, which belongs to the same subfamily 6 as *SibHLH7* and has a similar motif composition, can interact with 5′-GAGA-3′ cis regulatory elements in vitro, and activates the transcription of planar *AtPGR* [70]. This enhances the resistance to abiotic stress. Similarly, the expression of *SibHLH7* was significantly upregulated in almost all abiotic stresses, which may enhance the adaptability of foxtail millet to the environment in a similar manner. In addition, *AtbHLH45* (*At3g06120*), *AtbHLH97* (*At3g24140*), and *AtbHLH98* (*At5g53210*) belong to three subfamilies related to stomatal development in leaves [71, 72]. Under drought conditions, *SibHLH1* expression was rapidly and significantly upregulated in leaves and stems, which may help regulate stomatal action to reduce water loss. This function is consistent with the physiological characteristics of foxtail millet, which is a drought-tolerant plant. As expected, *SibHLH1* expression levels gradually decreased in the dark, which may contribute to stomatal dilation and increased respiration. These expression pattern results suggest that *bHLH*-TFs are involved in a complex cross-regulatory network. For example, *SibHLH*29 and *SibHLH*30 showed a significant positive correlation, and they had a strong response to alkali, NaCl, and dark exposure, suggesting that they have a synergistic regulatory effect under various adverse conditions.

## Conclusion

We first identified and analysed the genome-wide *SibHLH* family in *S. italica.* These 187 genes were divided into 21 groups and one UC group. Segment and tandem duplications contributed to the expansion of the *SibHLH* family, and segment duplication may have a more important contribution. We analysed the expression of these genes in different tissues and different fruit development stages during the filling period, in addition to their response to eight abiotic stresses. Based on the above analyses of genetic structures and functional speculation in the *SibHLH* family, some key candidate genes were preliminarily screened out, such as *SibHLH7*, *SibHLH22*, and *SibHLH36*. These results indicated that the *bHLH* transcription factors in *S. italica* may be involved in various physiological processes of plant growth and development. The collective data provides a reference for future studies of *bHLH* genes on foxtail millet.

## Methods

### Gene identification

We downloaded the entire foxtail millet genome from the Ensembl Genomes website (http://ensemblgenomes.org/). Based on two BLASTp methods [73, 74], *bHLH* family members were identified. First, the candidate bHLH proteins of foxtail millet were authenticated by a BLASTp search. Second, the hidden Markov model (HMM) file corresponding to the *bHLH* domain (PF00011) was downloaded from the Pfam protein family database (http://pfam.sanger.ac.uk/). The bHLH protein sequences of foxtail millet were aligned using the HMM model in HMMER3.0, with a cut-off of 0.01 (http://plants.ensembl. org/hmmer/index.html) [75]. The existence of *bHLH* core sequences was verified using the PFAM and SMART programs (http://smart.embl-heidelberg.de/) [76, 77]. The 187 *SibHLH* genes were identified in the foxtail millet genome. Then, 187 SibHLH proteins were used as initial queries in the NCBI protein database (https://blast.ncbi.nlm.nih.gov/Blast.cgi? PROGRAM = blastp&PAGE_TYPE = BlastSearch&LINK_LOC = blasthome) using BLASTp to verify bHLH proteins. Finally, the basic features of the trihelix proteins of the *bHLH* genes of *S. italica* (sequence length, MW, pI, subcellular localisation) were identified using the ExPasy (http://web.expasy.org/protparam/).

### *bHLH* gene structure and conserved motif analysis

ClustalW was used to create a multi-sequence alignment project with default parameters to further analyse the characteristic domain of the SibHLH proteins [78]. Mega software (version 7.0) and GeneDoc2.7 (http://genedoc.software.informer.com/2.7/) were then used to manually adjust the *bHLH* structure domain using the deduced amino acid sequences. The gene structure display server (GSDS; http://gsds.cbi.pku.edu.cn) online program [79] was used to analyse the exon-intron structures of the *SibHLH* genes. The conserved motifs in the encoded bHLH proteins were studied, to investigate their structural differences. The MEME online program (http://meme.nbcr. net/meme/intro. html) was used to analyse the motifs of the SibHLH proteins [80, 81]. The optimised parameters included a maximum number of motifs of 10 and an optimum width of 6 to 200 residues [81, 82].

### Chromosomal distribution and gene duplication

All *SibHLH* genes were mapped to locations on the *S. italica* chromosomes based on physical location information. The Circos program was used to process the chromosomal location information of the *SibHLH* genes [83]. Analysis of *SibHLH* gene replication events were analysed using multiple collinear scanning toolkits (MCScanX). The homology of the *bHLH* genes between *S. italica* and six other plants (*A. thaliana*, *S. lycopersicum*, *S. tuberosum*, *B. distachyon*, *O. sativa* subsp. *indica,* and *Z. mays*) was analysed using the dual synteny plotter (https://github.com/CJ-Chen/TBtools).

### Phylogenetic analysis and classification of *SibHLH* family

According to the classification of bHLH proteins of Arabidopsis, 187 bHLH proteins of *S. italica* were divided into several groups. In MEGA 7.0, the NJ tree was constructed used the Jukes–Cantor model. The phylogenetic tree was constructed with a bootstrap value of 1000 and assigned with Geneious R11 with BLOSUM62 cost matrix. In addition, a multi-species phylogenetic evolutionary tree was constructed that included SibHLH proteins and bHLH protein sequences of six plants (*A. thaliana*, *S. lycopersicum*, *S. tuberosum*, *B. distachyon*, *O. sativa* subsp. *indica,* and *Z. mays*), downloaded from the UniProt database (UniProt: https://www.uniprot.org/).

### Plant materials, growth conditions, and abiotic stress in *S. italica*

*S. italica* cv. Yugu 1*,* a typical cultivated variety in northern China, was used throughout the study. Since 2020, ‘Yugu 1’ has grown in the greenhouse of the experimental base located at the Guizhou University farm. In the appropriate development stages of foxtail millet, we obtained the flag leaves, third leaves, roots, stems, fruits, and three developmental stages of fruits and glumes at 18 (green fruit stage), 25 (discolouration stage), and 32 (initial maturity stage) DPA in the laboratory. All organ samples were taken from five plants under the same growth conditions, quickly placed in liquid nitrogen, and stored at − 80 °C until further use. In addition, the expression patterns of *SibHLH* genes under different stresses were studied, and the expression levels of 15 *SibHLH* genes were analysed. All seedlings of *S. italica* were planted in seedling trays, and 50 mL of solution was poured into each independent tray to fully immerse the root system. At the seedling stage (28 days), the plant seedlings of *S. italica* were subjected to eight different abiotic treatments: acid (HCl 0.1 M), alkali (NaOH 0.2 M), salt (5% NaCl), flooding (whole plant), drought (30% PEG6000), darkness (complete shading), heat (40 °C), and cold (4 °C). Each stress treatment was performed with five replicates, and the leaves, roots, and stems were taken at 0, 2, and 24 h for qRT-PCR analysis.

### Total RNA extraction, cDNA reverse transcription, and qRT-PCR analysis

Total RNA was extracted using the plant RNA extraction kit (TaKaRa Bio) and treated with RNase-free DNase I to remove trace amounts of DNA. The qRT-PCR primers were designed using Primer 5.0 software (Additional file 6: Table S6). Using *ACTIN* (*Si001873m.g*) as an internal control, standard RT-qPCR with SYBR Premix Ex Taq II (TaKaRa Bio) was repeated at least three times on a CFX96 Real-Time System (Bio-Rad). The total reaction system was 20 μL, contains 1 μL cDNA (100 ng/μL^− 1^), 10 μL SYBR Green Realtime PCR Master Mix, 0.5 μL forward and reverse primers, 8 μL rnase-free water, respectively. The reaction procedures for real-time quantitative PCR detection were as follows: pre-denaturation at 95 °C for 30s, denaturation at 95 °C for 5 s, annealing at 60 °C for 20s, and extension at 72 °C for 20s, with a total of 40 cycles. All primers were validated by melting curve analysis. The experimental data was calculated using the 2^− (ΔΔCt)^ method [84].

### Statistical analysis

We processed and analysed all the above data via variance analysis using JMP6.0 software (SAS Institute). The means were compared using the least significant difference test at significance levels of 0.05 and 0.01. The histogram was drawn using Origin 8.0 software .

## Supplementary Information

**Additional file 1: Table S1.** List of 187 *S. italica bHLH* genes identified in this study.

**Additional file 2: Table S2.** Analysis and distribution of conserved motifs in *S. italica bHLH* proteins.

**Additional file 3: Table S3.** Tandem duplication events in *S. italica bHLH* genes.

**Additional file 4: Table S4.** The 38 pairs of segmental duplications in *S. italica bHLH* genes.

**Additional file 5: Table S5.** One-to-one orthologous genes relationships between *S. italica* and other plants.

**Additional file 6: Table S6.** Primer sequences for qRT-PCR.

**Additional file 7: Fig. S1.** The correlations 15 *S. italica bHLH* genes in several plant organs. Positive number: positively correlated; negative number: negatively correlated. Red numbers indicate a significant correlation at the level of 0.05.

**Additional file 8: Fig. S2.** The correlations 15 *S. italica bHLH* genes in several abiotic stresses.

## Data Availability

The entire *Setaria italica* genome sequence information was obtained from the Ensembl Genomes website (http://ensemblgenomes.org/). *S. italica* materials (Yugu 1) used in the experiment were supplied by Prof. Cheng Jianping of Guizhou University. The datasets supporting the conclusions of this study are included in the article and its additional files.

## Abbreviations

*bHLH*: Basic helix-loop-helix
*SibHLH*: *Setaria italica bHLH*
qRT-PCR: Quantitative real-time polymerase chain reaction
At*bHLH*: *Arabidopsis thaliana bHLH*
HMM: Hidden Markov Model
pI: Isoelectric point
LG: Linkage group
