# Supplementary figures and images for: Genome-wide identification and expression analysis of the bHLH transcription factor family and its response to abiotic stress in foxtail millet (Setaria italica L.)
Source: BMC Genomics. 2021 Oct 30;22:778. doi: 10.1186/s12864-021-08095-y (PMC8557513; doi:10.1186/s12864-021-08095-y)

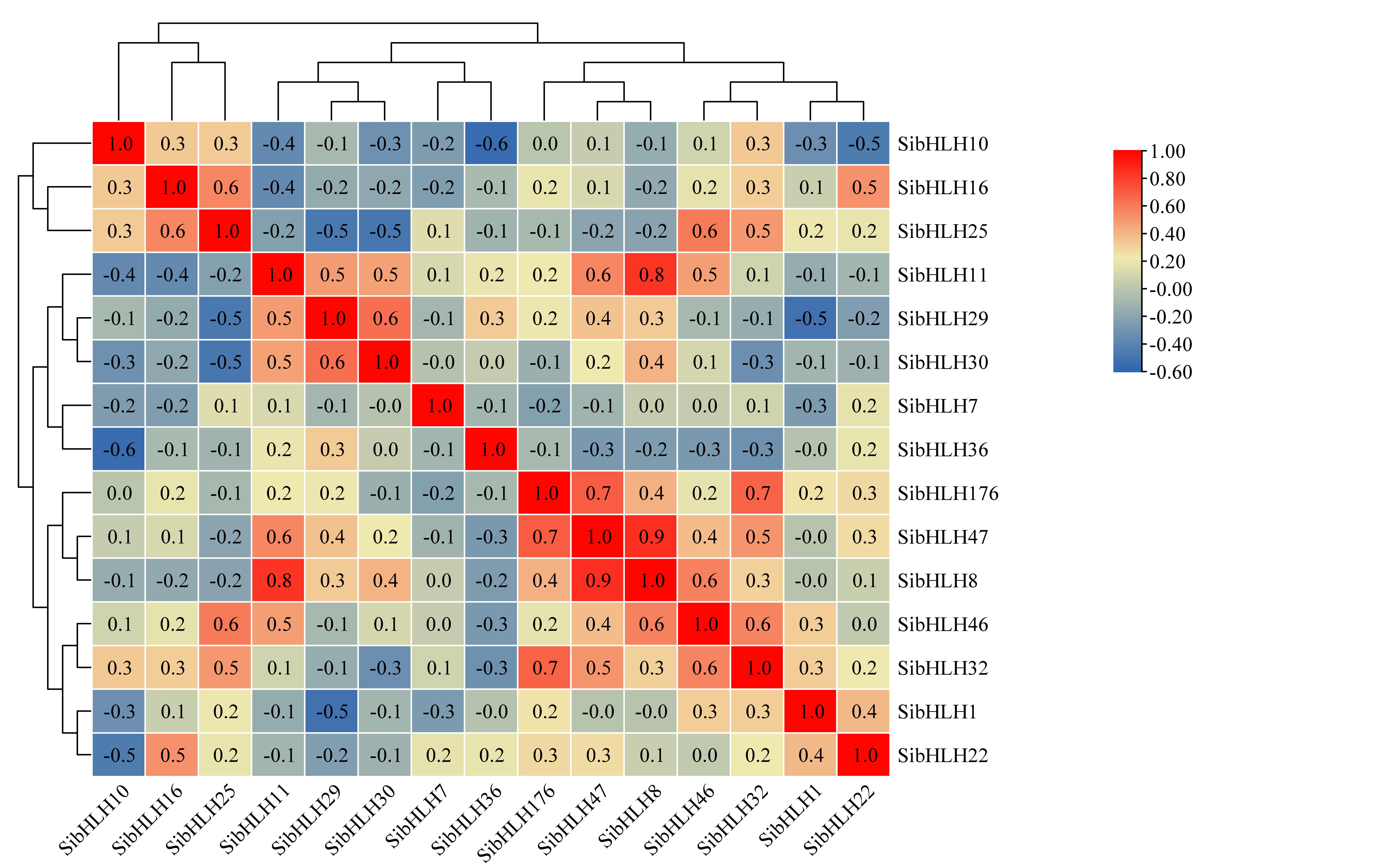

Supplement: Supplementary file 7 — Additional file 7: Fig. S1. The correlations 15 S. italica bHLH genes in several plant organs. Positive number: positively correlated; negative number: negatively correlated. Red numbers indicate a significant correlation at the level of 0.05. [file 12864_2021_8095_MOESM7_ESM.jpg]

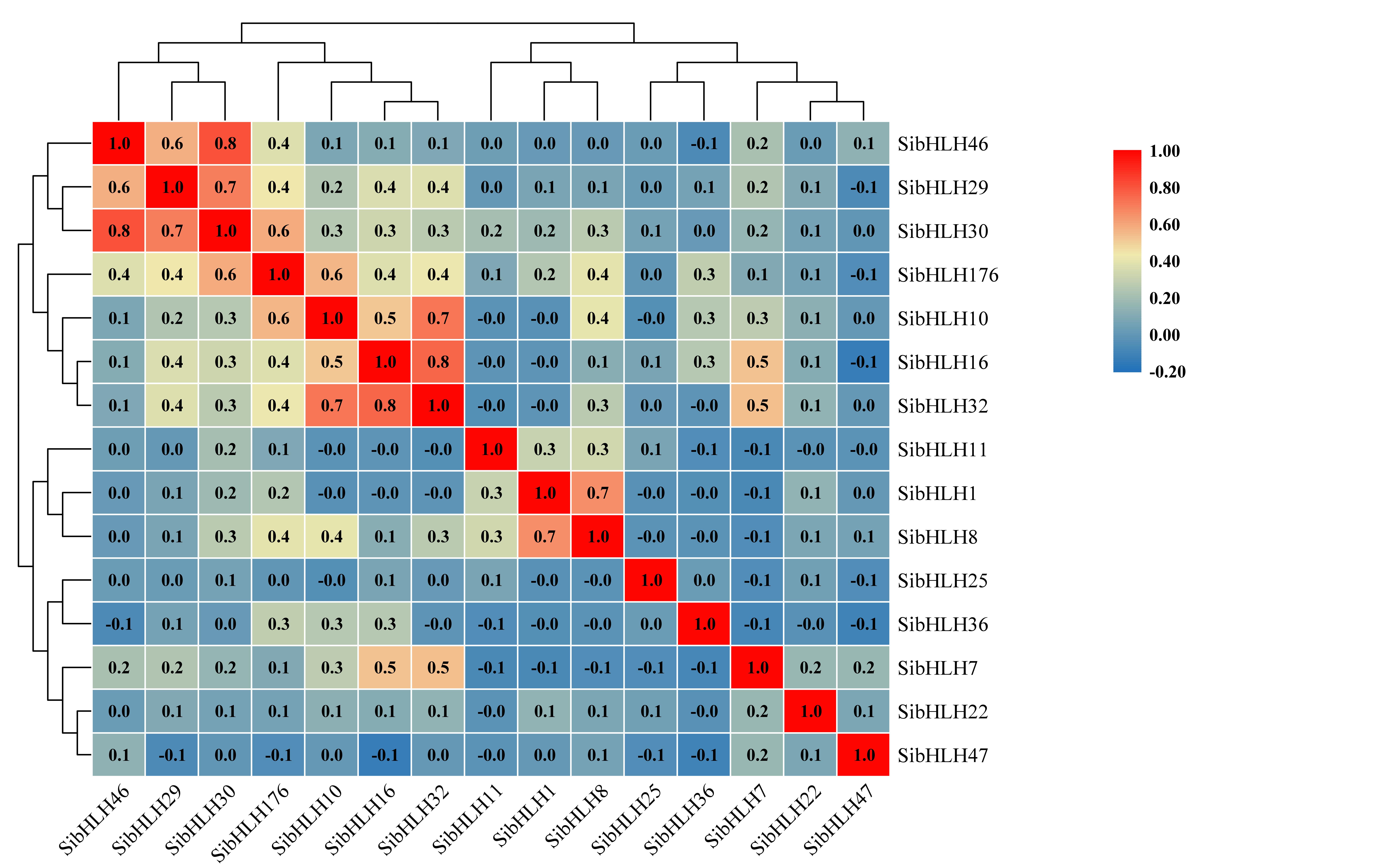

Supplement: Supplementary file 8 — Additional file 8: Fig. S2. The correlations 15 S. italica bHLH genes in several abiotic stresses. [file 12864_2021_8095_MOESM8_ESM.jpg]
